# O.5.1-10 Promoting mobility among wellbeing employees in Lahti, Finland

**DOI:** 10.1093/eurpub/ckad133.240

**Published:** 2023-09-11

**Authors:** Riitta-Maija Hämäläinen

**Affiliations:** Wellbeing services county of Päijät-Häme, Finland; riitta-maija.hamalainen@paijatha.fi

## Abstract

Versatile everyday physical activity outdoors increases the wellbeing of people of all ages and prevents illnesses. The amount of physical activity among working-age people affects coping and recovery at work and wellbeing after work. A healthy, safe, and sustainable living environment supports everyday physical activity and mobility outdoors. Within the NatureStep to Health programme 2022-2032 the Wellbeing services county of Päijät-Häme, Lahti, Finland promotes mobility among employees to in-crease nature contacts and to improve health.

The Wellbeing services county of Päijät-Häme piloted sustainable mobility among employees. The county employs approximately 7600 persons, of which 89% are women, for health, social and rescue services. The Wellbeing services county of Päijät-Häme aimed to build recommendations to provide occupational benefit packages for employees. The pilots were expected to provide information on short-term, intermediate, and long-term outcomes for planning, developing and selecting further actions on sustainable mobility.

The pilots focused on how employer provided sustainable mobility initiatives were adopted by employ-ees. The pilots included employer-subsidized commuter tickets for local transport, employer-provided bicycle benefits and shared city e-bikes and e-scooters during the working day covered by employer. The employer-subsidized commuter tickets are personal tickets meant for commuting free of taxes up to 3 400 €/year. An employer-provided bicycle benefit is a bicycle intended for the employeés personal use for commuting by tax-deductible income up to 1 200€/year.

Despite the short pilot approach to increase mobility and the limited number of participants, they were loyal and eager to continue the provided mobility modes after the pilot period. The participants' com-muting distances were short to switch to alternative modes of transport in a strongly female-dominated workplace.

The pilot confirms that sustainable mobility supports employees’ wellbeing, increase physical activity, and decrease the costs of commuting spilling over to increase free time mobility as well. Employers have an active role in both implementing sustainable mobility incentives and in supporting the continua-tion by human resource policies. The maintenance of mobility requires appropriate facilities, such as safe and covered well-located bicycle parking, dressing rooms, lockers, and showers. The initiative shows employer's commitment to the mobility shift and corporate responsibility.

